# AGING AND HEALTH IN THE CARIBBEAN REGION: AN OVERVIEW OF THE STATE OF RESEARCH

**DOI:** 10.1093/geroni/igad104.0511

**Published:** 2023-12-21

**Authors:** Nekehia Tamara Quashie

**Affiliations:** University of Rhode Island, Kingston, Rhode Island, United States

## Abstract

The Caribbean region is undergoing rapid population aging. While there is diversity in the pace of population aging among countries within the region, there are some shared population health challenges including the increasing prevalence of chronic non-communicable diseases and the inequalities therein. Relatedly, many Caribbean countries do not have well-established long-term care systems. Therefore, older adults primarily rely on informal care and support mainly from family members, which has implications for the health of older adults and their caregivers. Although Caribbean populations are understudied within gerontology, there is a growing body of research. This presentation provides an overview of the existing state of research on the social determinants of health among older adults within Caribbean societies and discusses directions for future research.

